# Substrates of neuropsychological functioning in stimulant dependence: a review of functional neuroimaging research

**DOI:** 10.1002/brb3.65

**Published:** 2012-06-26

**Authors:** Cleo L Crunelle, Dick J Veltman, Jan Booij, Katelijne Emmerik – van Oortmerssen, Wim den Brink

**Affiliations:** 1 Amsterdam Institute for Addiction Research and Department of Psychiatry, Academic Medical Center, University of AmsterdamAmsterdam, The Netherlands; 2 Department of Nuclear Medicine, Academic Medical Center, University of AmsterdamAmsterdam, The Netherlands; 3 Department of Psychiatry, Vrije Universiteit medical centerAmsterdam, The Netherlands; 4 Arkin Mental Health and Addiction Treatment CentreAmsterdam, The Netherlands

**Keywords:** Addiction, fMRI, functional imaging, magnetic resonance imaging, stimulant dependence, stimulants

## Abstract

Stimulant dependence is associated with neuropsychological impairments. Here, we summarize and integrate the existing neuroimaging literature on the neural substrates of neuropsychological (dys)function in stimulant dependence, including cocaine, (meth-)amphetamine, ecstasy and nicotine dependence, and excessive caffeine use, comparing stimulant abusers (SAs) to nondrug using healthy controls (HCs). Despite some inconsistencies, most studies indicated altered brain activation in prefrontal cortex (PFC) and insula in response to reward and punishment, and higher limbic and anterior cingulate cortex (ACC)/PFC activation during craving and attentional bias paradigms in SAs compared with HCs. Impulsivity in SAs was associated with lower ACC and presupplementary motor area activity compared with HCs, and related to both ventral (amygdala, ventrolateral PFC, insula) and dorsal (dorsolateral PFC, dorsal ACC, posterior parietal cortex) systems. Decision making in SAs was associated with low dorsolateral PFC activity and high orbitofrontal activity. Finally, executive function in SAs was associated with lower activation in frontotemporal regions and higher activation in premotor cortex compared with HCs. It is concluded that the lower activations compared with HCs are likely to reflect the neural substrate of impaired neurocognitive functions, whereas higher activations in SAs compared with HCs are likely to reflect compensatory cognitive control mechanisms to keep behavioral task performance to a similar level as in HCs. However, before final conclusions can be drawn, additional research is needed using neuroimaging in SAs and HCs using larger and more homogeneous samples as well as more comparable task paradigms, study designs, and statistical analyses.

## Introduction

Substance abuse is characterized by recurring compulsive urges to use drugs, despite long-term negative consequences, which may include a wide range of psychological, social, and medical complications. Moreover, even after treatment and regardless of motivation to quit, relapse is common. In 2008 alone, over 700,000 people in Europe and over 3.5 million people in the United States were seeking treatment for problematic drug use (World Drug Report 2008).

Several theories for drug dependence have been presented over the years, including drug use as an alleviation from distress or drug withdrawal (*negative reinforcement theory* [Hull 1943; Khantzian 1985; Koob and Moal 2008]) and drug use as a positive reinforcer, that is, to increase and maintain pleasure (*positive reinforcement theory* [Stewart et al. 1984]). However, euphoric positive effects do not seem to persist in humans after years of compulsive drug use and none of these models has yet satisfactorily explained maintenance of compulsive drug use and the urge to continue drug use, often despite a strong motivation and serious attempts to become and remain abstinent. As a possible solution, the *Incentive-Sensitization Theory* was introduced as a neuroadaptational model in which various neurobiological changes pave the way to persistent drug use behavior and craving (Robinson and Berridge 1993). The *Impaired Response Inhibition and Salience Attribution* (I-RISA) model of Goldstein and Volkow (2002) conceptualized drug dependence as a cognitive and emotional process associated with a dual process of overvaluation of drug rewards and undervaluation of natural reinforcers, due to limbic dysregulation (impaired salience attribution) together with inhibitory deficits due to prefrontal impairment (impaired response inhibition). Accordingly, compulsive drug use would result from poorly developed (prefrontal) reflective processes dependent on executive functioning, taken over by a fast motivational (amygdalar) impulse process (Bechara 2005; Wiers et al. 2007). This model integrates behavioral, emotional, and cognitive processes and thereby expanded the traditional concepts that relied on positive and negative reinforcement for compulsive drug use and relapse. In addition to the I-RISA model, the *Habitual Behavioral Model* emphasizes the importance of a switch from goal-directed behavior to habitual behavior during the development of drug dependence. Habitual behavior would be less sensitive to outcome values and would lead to loss of voluntary control and the development of compulsive behavior, such as compulsive drug use. The switch to habitual behavior would represent a progression from prefrontal cortical to striatal control and a switch from ventral to more dorsal striatal regions (Wood and Neal 2007; Everitt et al. 2008). Whether changes in neuropsychological functioning should be viewed as a vulnerability trait or a response to chronic drug abuse still needs to be elucidated. Several studies have provided evidence for the involvement of predisposing genetic and environmental factors (Morgan et al. 2002a; Bevilacqua and Goldman 2009), while others have described similar neurobiological changes as a response to chronic drug use (Nader et al. 2002; Volkow et al. 2004), or have assumed that both processes are present and mutually enhancing (Nader et al. 2006).

While early hypotheses were stated from a behaviorist and psychological point of view (Hull 1943), subsequent theories were increasingly based on neurobiological animal research. With time, studies focused on integrating results from animal and human studies, and neuroanatomical substrates and dysregulated neurotransmitter systems were hypothesized to underlie the motivation to administer drugs, while recognizing the important role of genetic along with social factors as contributors in the pathophysiology of drug use and addiction. Importantly, recent models of addiction have increasingly incorporated neuropsychological aspects of drug dependence, aided by the rapid expansion of the field of functional neuroimaging (for a review on substrates and neurocircuitries considered important in drug dependence, see the recent reviews of Goldstein et al. 2009a; Koob and Volkow 2010). However, results of these imaging studies usually do not allow causal inferences to be made, which should also be kept in mind when reading this review.

So far, research seems to indicate that stimulant dependence (cocaine, amphetamine, methamphetamine, 3,4-methylenedioxymethamphetamine [MDMA, main com-ponent of ecstacy]), and – to a lesser extent – nicotine and caffeine are associated with more severe neuropsychological impairments than alcohol, cannabis, or even opioid dependence (for a review, see Holst and Schilt 2011). For example, amphetamine and cocaine abusers performed worse on verbal memory, abstraction ability, and on mathematic skills compared with matched alcohol and polydrug abusers (Block et al. 2002). Moreover, amphetamine abusers were more impaired in planning ability (Ersche et al. 2006) and decision making (Rogers et al. 1999) than opiate abusers. Finally, a recent study showed that abstinent polysubstance abusers with cocaine as their primary drug of choice were more impaired on measures of inhibitory control, cognitive flexibility and working memory than abstinent polysubstance abusers with heroin as their primary drug of use (Verdejo-Garcia and Perez-Garcia 2007).

The aim of the present review is to summarize and integrate the existing literature on the neuroanatomical substrates associated with neuropsychological impairments in stimulant dependence. The review is organized according to the various neuropsychological functions that are considered relevant for the development and/or maintenance of drug dependence and involves several distinct neural circuits (e.g., Volkow et al. 2004): Reward and punishment processing (Section 1); Cue-reactivity and attentional bias (Section 2); Impulsivity (Section 3); and Decision making and executive function (Section 4). Each section starts with a brief description of the neuropsychological function with commonly used tasks followed by behavioral data from these neuropsychological tasks in stimulant abusers (SAs) compared to healthy controls (HCs), and completed by a summary and discussion of functional neuroimaging studies in SAs compared to HCs.

## Literature Search

A literature search was performed using Pubmed and Embase until June 2011 with the key search terms including the neuropsychological tasks, cocaine-related disorders, amphetamine related disorders, substance related disorders, tobacco use disorders, *N*-methyl-3,4-methylenedioxyamphetamine, caffeine, magnetic resonance imaging (MRI), and positron emission tomography (PET). Functional MRI (fMRI) uses blood oxygenation level dependent (BOLD) contrast to visualize differences in regional brain activity, a technique with much higher temporal and higher spatial resolution than PET. Before the introduction of fMRI, [^15^O] PET was widely used to perform activation studies due to the relatively short half-life of ^15^O (122 s), permitting repeated task versus baseline scans during a single session. In contrast, the ^18^F-tracer fluorodeoxyglucose has a much longer half-life (about 110 min) and is therefore primarily used for resting-state studies. The latter were omitted from this review, as were single photon emission computerized tomography (SPECT) studies. Electroencephalography (EEG) studies were also excluded, because of inherent poor spatial resolution. Finally, diffusion tensor imaging provides a visualization of white matter tracts by measuring voxel-based diffusion coefficients of water in brain tissue and fDTI is based on the idea that changes in white matter axonal volume may accompany brain activation patterns (Mandl et al. 2008). However, fDTI is a highly novel technique that has not been adequately validated and these studies were also excluded. This review thus focuses on fMRI and [^15^O] PET studies.

Searches yielded 107 articles, of which only 40 used functional neuroimaging. Another 27 potentially relevant articles were found through cross-referencing. Of these 67 articles, 37 were excluded because they included only polydrug users (*n* = 3), did not have a matched control group (*n* = 16), re-used an external (nonmatched) control group from a previous study (*n* = 2), did not match for alcohol and/or cannabis use (*n* = 7), or included other imaging techniques (*n* = 9).

This review thus includes 26 studies using fMRI and four studies using [^15^O] PET. The most frequently studied substance was cocaine (*n* = 17), followed by nicotine (*n* = 5), (meth-)amphetamine (*n* = 4), and ecstasy (*n* = 4). No studies were found in subjects with excessive use of caffeine compared with low or no caffeine consumers. For several details concerning the reviewed studies (e.g., neuroimaging technique, task, abused drug, time since last use, sample size, and summary of findings), four tables (Tables 1–4) are presented in the subsequent sections.

**Table 1:** Overview of the selected reviewed studies on reward and punishment processing in stimulant abusers versus healthy controls

	de Ruiter et al. (2009)	Goldstein et al. (2007a,b)
Technique	fMRI	fMRI
Task	PRLT	monetary reward
Abused drug	Nicotine	Cocaine
Drug use (SD)	–	17.6 years (6.7 years)
Time since abstinence	Current users	1–90 days
Sample size – users (% male)	19 (100%)	16 (75%)
Sample size – HCs (% male)	19 (100%)	13 (70%)
Behavioral findings (vs. HCs)	↓ Accuracy/gains	↓ Accuracy/gains
	= RT	= RT
Response during gains (vs. HCs)	↑ PFC	↓ OFC
	↑ Insula	↓ Cerebellum
	↑ Parietal regions	
Response during losses (vs. HCs)	↓ VL PFC	–

fMRI, functional magnetic resonance imaging; PRLT, probabilistic reversal learning task; SD, standard deviation; HC, healthy control; RT, reaction time; VL PFC, ventrolateral prefrontal cortex; OFC, orbitofrontal cortex.

**Table 2:** Overview of the selected reviewed studies using cue-reactivity paradigms in stimulant abusers versus healthy control subjects

	Maas et al. (1998)	Childress et al. (1999)	Garavan et al. (2000)	Wexler et al. (2001)	Due et al. (2002)	David et al. (2005)	Okuyemi et al. (2009)	Goldstein et al. (2009a,b)	Goudriaan et al. (2010)	Wilcox et al. (2011)
Technique	fMRI	[^15^O] PET	fMRI	fMRI	fMRI	fMRI	fMRI	fMRI	fMRI	fMRI
CR stimulus type	Audiovisual	Audiovisual	Audiovisual	Audiovisual	Visual	Visual	Visual	Verbal	Visual	Audiovisual
Abused drug	Cocaine	Cocaine	Cocaine	Cocaine	Nicotine	Nicotine	Nicotine	Cocaine	Nicotine	Cocaine
Mean drug use	>6 months	8.1 years	11 years	Unknown	Unknown	Unknown	19 years	16.5 years	Unknown	Chronic
Time since last use	Unknown	13.5 days	Unknown	>14.9 days	>12 h	>12 h	25 min	4.6 days	16–18 h	>3 days
Sample – users, *N* (% male)	6 (100%)	14 (100%)	17 (82%)	11 (73%)	11 (64%)	9 (44%)	17 (35%)	17 (76%)	18 (100%)	14
Sample – HCs, *N* (% male)	6 (100%)	6 (100%)	14 (36%)	21 (38%)	6 (67%)	11 (27%)	17 (29%)	17 (82%)	17 (100%)	16 (Matched)
CR craving (vs. prescan)	↑	↑	↑	↑	↑	=	Unknown	↑	=	↑
CR questionnaire	VAS	10 items	Likert scale	VAS	3 items	5 items	QSU-brief	5 items	QSU	CCQ
CR response (vs. HCs)									FTND > 5	
	↑ ACC	↑ ACC	↑ Prefrontal	↑ ACC	↑ Amygdala	↑ NcA	↑ VL PFC	↓ ACC	↑ r ACC	↑ DLPFC
	↑ DL PFC	↑ Amygdala	↑ Insula	↓ Prefrontal	↑ Hippocampus		↑ m PFC		↑ m PFC	↑ Occipital
		↓ Caudate	↑ Cingulate	↓ Temporal	↑ VTA		↑ OFC		↑ Insula	
					↑ Thalamus		AA > CC		↑ Temporal	
					↑ IPS		↑ Amygdala			
					↑ Frontal gyrus		↑ Caudate			

fMRI, functional magnetic resonance imaging; PET, positron emission tomography; CR, cue-reactivity; HC, healthy control; VAS, visual analog scale; QSU, questionnaire for smoking urges; CCQ, cocaine craving questionnaire; FTND, Fagerström test for nicotine dependence; AA, Afro-American; CC, Caucasian; (r) ACC, (rostral) anterior cingulated cortex; (DL/VL/m) PFC, (dorsolateral/ventrolateral/medial) prefrontal cortex; VTA, ventral tegmental area; IPS, intraparietal sulcus; NcA, nucleus accumbens; OFC, orbitofrontal cortex.

**Table 3:** Overview of the selected reviewed articles on motor and cognitive impulsivity in substance abusers compared to nondrug using control participants

	Motor impulsivity						Cognitive impulsivity		
		
	Kaufman et al. (2003)	Hester and Garavan (2004)	Li et al. (2008)	Bolla et al. (2004)	Hanlon et al. (2009)	Karageorgiou et al. (2009)	Monterosso et al. (2007)	Hoffman et al. (2008)	Meade et al. (2011)
Technique	fMRI	fMRI	fMRI	[^15^O] PET	fMRI	fMRI	fMRI	fMRI	fMRI
Task	Go/No-go	Go/No-go	Stop-Signal	STROOP	Finger-tapping	Finger-tapping	DD	DD	DD
Abused drug	Cocaine	Cocaine	Cocaine	Cocaine	Cocaine	MDMA	MA	MA	Cocaine
Mean drug use	11.2 years	14 years	10.2 years	>2 years	16.3 years	Unknown	>1 year	>1 year	20.3 years
Time since last use (SD)	18–72 h	12–72 h	>2 weeks	23 days	9–30 h	669 days (665)	Current users	2–8 weeks	Current users
Sample – users (% male)	13 (62%)	15 (60%)	15 (100%)	13 (unknown)	14 (57%)	14 (71%)	12 (67%)	19 (68%)	15 (73%)
Sample – HCs (% male)	14 (29%)	15 (47%)	15 (100%)	13 (unknown)	14 (57%)	10 (50%)	17 (71%)	17 (71%)	11 (81%)
Performance (vs. HCs)	↓	↓	=	=	↓	=	↑ DD	↑ DD	↑ DD (ns)
Response (vs. HCs)	↓ DL PFC	↓ ACC*	↓ rACC*	↓ Left ACC	↓ Caudate	↑ SMA	↓ DL PFC	↓ Caudate	↓ PFC
	↓ ACC*	↓ Pre-SMA*		↓ PFC	↓ Putamen		↓ IPS	↓ Precuneus	↓ ACC
	↓ Parietal	↓ PFC*		↑ Right ACC				↓ ACC	
	↓ Putamen							↓ DL PFC	
	↓ Pre-SMA*								
	↓ Insula*								

fMRI, functional magnetic resonance imaging; PET, positron emission tomography; DD, delayed discounting; MA, methamphetamine; SD, standard deviation; HC, healthy control; (r)ACC, (rostral) anterior cingulated cortex; (DL) PFC, (dorsolateral) prefrontal cortex; IPS, intraparietal sulcus; SMA, supplementary motor area.

* Brain responses during inhibitory trials; ns, nonsignificant.

**Table 4:** Overview of the selected reviewed articles on decision making and executive control

	Decision making			Memory				Flexibility, attention, and planning		
			
	Bolla et al. (2003)	Ersche et al. (2005)	Paulus et al. (2003)	Daumann et al. (2003a)	Daumann et al. (2003b)	Jacobsen et al. (2004)	Bustamante et al. (2011)	Kubler et al. (2005)	de Ruiter et al. (2009)	Goldstein et al. (2007b)
Technique	[^15^O] PET	[^15^O] PET	fMRI	fMRI	fMRI	fMRI	fMRI	fMRI	fMRI	fMRI
Task	IGT	CambrRT	2-CPT	N-back	N-back	N-back	N-back	Switch task	PRLT/ToL	SAT
Abused drug	Cocaine	Amphetamine	MA	Ecstasy	Ecstasy	Ecstasy	Cocaine	Cocaine	Nicotine	Cocaine
Mean drug use	>2 years	16 years	17 years	53/16 month	>2 years	<2 years	11.7 years	2.5–18 years	Unknown	17.6 years
Time since last use	25 days	Current users	6–46 days	89/330 days	23 days	Unknown	>4–5 days	0–3 days	10 h	0–90 days
Sample – users (% male)	13 (77%)	15 (73%)	14 (100%)	*2x* 11 (73%)	8 (50%)	6 (33%)	15 (100%)	8 (unknown)	19 (100%)	16 (75%)
Sample – HCs (% male)	13 (77%)	15 (73%)	14 (71%)	11 (73%)	8 (50%)	6 (33%)	15 (100%)	8 (unknown)	19 (100%)	12 (67%)
Performance (vs. HCs)	↓	=	=	=	=	=	=	=	↓/=	=
Task response (vs. HCs)	↑ OFC	↓ DL PFC	↑ OFC	↑ Parietal	↑ Premotor	↑ HippoC	↓ Parietal	↓ Cingulated	*PRLT:*	↓ ACC
	↑ Putamen	↑ OFC	↑ DL PFC	↓ Frontal	↓ Temporal			↓ Frontal	↓ VL PFC	↓ DL PFC
	↑ PCG		↑ Parietal	↓ Temporal				↓ Thalamus	↓ Premotor	↓ VM PFC
	↓ DL PFC			↓ ACC				↓ Putamen	↑ Insula	↓ Frontal
	↓ m PFC							↓ Precuneus	↑ Frontal	Gyrus
	↓ Parietal								Operculum	↓ Cerebellum
	↓ Cerebellum								*ToL:*	↓ (Pre)cuneus
									↓ Parietal	↓ Thalamus

fMRI, functional magnetic resonance imaging; PET, positron emission tomography; IGT, Iowa Gambling Task; CambrRT, Cambridge Risk Task; 2-CPT, 2-choice prediction task; PRLT, probabilistic reversal learning task; ToL, Tower of London; SAT, sustained attention task; MA, methamphetamine; HC, healthy controls; OFC, orbitofrontal cortex; PCG, postcentral gyrus; (DL/VL/m/VM) PFC, (dorsolateral/ventrolateral/medial/ventromedial) prefrontal cortex; HippoC, hippocampus.

## Results and Discussion

### Section 1: Reward and punishment processing in stimulant dependence

#### Task paradigms and behavioral findings during reward and punishment processing

Reduced sensitivity for reward or punishment, or negative affect, is hypothesized to cause persistent drug-taking behavior by reducing aversive states (Baker et al. 2004) or by inducing lowered self-control (Segarra et al. 2000). With regard to addictive disorders, we like to notice that altered sensitivity to both natural reinforcers (this section) and drug (related) cues (next section) was found.

Sensitivity for reward and punishment of natural reinforcers can be measured using neurocognitive tasks with positive (monetary) feedback (reward) after a correct response or negative (monetary) feedback (punishment) following an incorrect response. Tasks that measure reward and punishment sensitivity include the stimulus-response learning task, and the probabilistic reversal learning task (PRLT), and a variety of gambling tasks which focus on processes like risk taking strategies regarding wins and losses, or on learning reward and punishment contingencies. The PRLT is a task in which the individual is required to adapt his or her response to changing contingencies (shifts) to win the largest amount of money. Tasks may feature several reward contingencies, representing high and low reward options or measure response differences during reward and punishment processing.

On the PRLT, cocaine abusers made fewer response shifts to changes in reward contingency than HCs, indicating high response perseveration in cocaine abusers (Ersche et al. 2008). Response perseveration is an important concept in addiction, because many drug dependent persons are not able to adapt their response to changing unforeseen events, such as the presentation of a drug-related reward, resulting in uncontrolled and compulsive drug use. In addition, response perseveration is of key importance in the treatment of drug dependence, where drug-addicted individuals need to learn how to change their automated responses following drug cues (i.e., cognitive–behavioral therapy). In most studies, response perseveration (compulsivity) was assessed with the PRLT, that is, a lack of adequate shifting following nonannounced punishment contingencies. Similarly, heavy smokers earned less money than HCs on the PRLT due to higher response perseveration in smokers (de Ruiter et al. 2009). Also, gambling tasks providing feedback with regard to gains and losses allow group comparisons of reward and punishment sensitivity. Subjects may choose between risky high reward and less risky lower reward options, and it is assumed that the choice of risky high rewards represents hypersensitivity to reward, hyposensitivity to punishment, or just risk taking behavior (Bechara et al. 2001; Clark and Robbins 2002; Tranel et al. 2002). Thus, whereas probabilistic reversal tasks necessitate flexible adaptation of behavior based on (monetary) contingencies, gambling tasks require the subject to devise a strategy that in the long run proves successful or focus on the level of risk taking with respect to rewards and losses. Cue-exposure tasks also involve (potentially) rewarding stimuli, but these are of a different nature, because they concern drug-related rather than more general natural rewards. In addition, cue-exposure tasks have a much lower cognitive demand and are, therefore, discussed separately in Section 2 (Attentional bias and craving).

#### Imaging reward and punishment processing: results and discussion

In a study by de Ruiter et al. (2009), heavy smokers showed higher activation in the right insula, right prefrontal cortex (PFC), and parietal regions bilaterally compared with HCs during monetary gain trials, indicating higher reward sensitivity, while showing significantly lower ventrolateral PFC activation compared with HCs during monetary loss trials, indicating lower punishment sensitivity in heavy smokers compared with HCs (de Ruiter et al. 2009). In cocaine abusers, however, lower overall brain activity was observed during reward trials compared with HCs, with significant lower activation in left orbitofrontal cortex (OFC) and left cerebellum (Goldstein et al. 2007a). Moreover, during high reward compared with no reward trials, HCs showed significant increases in activation in left OFC, lateral PFC, and mesencephalon, an effect that was not found in cocaine abusers. The authors suggested disrupted signaling between lateral PFC and OFC in cocaine abusers during monetary reward processing, implying lower sensitivity to monetary rewards in cocaine abusers (Goldstein et al. 2007a).

Thus it seems that the findings of Goldstein et al. (2007a) in cocaine users contradict those of de Ruiter et al. (2009) in heavy smokers, which might be due to differences in task paradigms (PRLT vs. monetary reward task), type of stimulant (cocaine vs. nicotine), and/or the duration of abstinence before the task (see Table 1 for a comparison overview between studies). Whereas both tasks include aspects of reward/punishment processing, they are very different in their original task requirements as the PRLT requires the individual to adapt his or her behavior several times to receive the reward, while the forced choice task requires the subject to adequately respond to certain trials while withholding their responses to other trials to obtain reward. Therefore, with regard to task differences, it should be noted that regional brain activation during rewarding stimuli may depend on several aspects of reward, such as reward expectation or the probability of receiving the reward, reward magnitude, and finally distancing from the reward. Additional studies using similar designs and experimental groups are needed to arrive at final conclusions regarding reward and punishment processing in SAs. However, together with the available behavioral studies, the current functional neuroimaging studies indicate that alterations in reward and punishment sensitivity in SAs may be (partly) responsible for ongoing drug use despite long-term negative consequences.

The findings from reward and punishment studies in SAs compared to HCs support the relevance of impaired prefrontal functioning in SAs proposed in addiction models with an important role for impaired evaluation of natural reinforcers (I-RISA model) and models with an important role for neurobiological changes in the PFC leading to persistent drug use (however, not necessarily as a cause as in the Incentive-Sensitization Theory).

### Section 2: Attentional bias and craving in stimulant dependence

#### Task paradigms and behavioral findings in attentional bias and craving

Attentional bias, craving, and relapse are presumably the most characteristic features of drug dependence. Drug abusers tend to direct their attention unconsciously to stimuli previously associated with drug use. Attentional bias may be due to enhanced sensitivity to drug-related rewards and constitutes a risk for the development of (physiological) cue-reactivity, which in turn may elicit craving, that is, a subjective feeling of intense need for the drug, which may ultimately lead to relapse (Field et al. 2009). To measure attentional bias in response to drug-related stimuli, an emotional Stroop task (the Drug Stroop) was developed, in which words or pictures related to drug use are shown in colors that have to be recognized and named by the participant. In its classic form, the Stroop task presents congruent stimuli (i.e., “red” printed in red ink) and incongruent stimuli (i.e., “green” in red ink) and measures interference between cognitive processes by requiring the participant to name the color (“red”) regardless of the word (“red” or “green”; Stroop 1935). It is hypothesized that the slower the speed of color-naming during incongruent stimuli, the more important the cognitive interference component. Consequently, in the Drug Stroop, the slower the speed of color-naming during stimuli associated with drug cues, the stronger the attentional bias toward the drug-related stimuli (Cox et al. 2006). For example, 24-h abstinent smokers showed higher attentional bias for smoking cues than current smokers (Waters and Feyerabend 2000). The Dot Probe task also measures attentional bias toward drug-related stimuli. Here, two stimuli (one drug-related and one neutral) are presented side by side, after which the images disappear and a dot appears for a short time. Fast responding toward the dot where a drug-related stimulus was previously shown is a measure for increased attentional bias. Smokers showed greater attentional bias toward smoking-cues than nonsmokers during a Dot Probe task (Ehrman et al. 2002). In addition, compared with current smokers, 12-h abstinent smokers showed increased attentional bias for smoking cues (Gross et al. 1993), and ex-smokers showed an intermediate level of attentional bias compared with current smokers and nonsmokers measured with the Dot Probe task (Ehrman et al. 2002). Using a related measure, abstinent crack-cocaine dependent patients had faster eye-movements toward cocaine-related pictures as compared to neutral pictures, and this correlated with self-reported intensity of cocaine craving (Rosse et al. 1997). It should be noted that the drug Stroop and the Dot Probe task both measure selective attention (i.e., to drug stimuli), but the Stroop task requires more cognitive effort and flexibility**,** which might be responsible for different findings when using these different paradigms.

Cue-reactivity is also an import aspect of drug addiction and refers to the physiological and related subjective reactions (craving) that occur in the presence of drug-related stimuli, and can ultimately lead to relapse. Cue-reactivity is generally investigated using a cue-exposure or cue-reactivity task. Unlike other neurocognitive tasks, cue-reactivity paradigms employed during functional imaging only require the participant to watch drug-related pictures or videos (without any cognitive effort), although some cue-reactivity tasks include easy binary tasks to control for attention differences, in which baseline trials are usually incorporated requiring similar motor responses.

#### Imaging attentional bias and cue-reactivity: results and discussion

To date, there are no neuroimaging studies on attentional bias in SAs, and therefore, the studies in this paragraph are restricted to those on cue-reactivity.

In an early study, Maas et al. (1998) found significantly higher activation in the anterior cingulate cortex (ACC) and left dorsolateral PFC (DLPFC) in crack-cocaine abusers compared with HCs. This was the first study that used a robust design (including HCs, a block design, and analyses following selected regions of interest [ROI]) and showed that fMRI was able to visualize craving in cocaine-dependent individuals, however, including important limitations such as the small sample size, the inclusion of cocaine-dependent individuals who were allowed to have a history of other drug use, and presenting of the visual analog scale (VAS) only twice (before and after the experiment), so that carry-over effects of craving across blocks could not be ruled out. Subsequently, Childress et al. (1999) showed higher regional cerebral blood flow (rCBF) in limbic structures (amygdala and anterior cingulate) and lower rCBF in basal ganglia (caudate) compared with HCs using [^15^O] PET. It should be noted that PET has lower spatial resolution than MRI, even when ROI are delineated on co-registered anatomical MRI scans, as in this study. Therefore, rCBF of the nucleus accumbens (NAcc) could not be assessed. A methodological problem was the small HC group (see Table 2), who were additionally significantly younger and higher educated than those in the cocaine-dependent group (Childress et al. 1999).

Whereas formal power calculations are problematic for [^15^O]-PET and fMRI, it has since been shown that in fMRI group sizes of at least 12 are required to reliably detect typical activations (Desmond and Glover 2002). Also, note that early imaging studies tend to report fixed-effects analyses, which limits generalizability of findings. The first fMRI study on cue exposure using an adequate sample was conducted by Garavan et al. (2000). Watching a cocaine video was associated with greater activation (compared with the neutral video) in a number of ROIs, including various prefrontal and limbic areas in cocaine abusers but not in HCs. The authors thus replicated the limbic activation found by Childress et al. (1999), concluding that cue-induced cocaine craving was primarily reflected by higher activation of prefrontal and limbic regions, that craving was not associated with a specific neuroanatomical substrate, but that cocaine users have a unique ability for learned, drug-related cues to produce similar brain activation patterns as potent, nondrug evocative stimuli in HCs. Furthermore, lower prefrontal and limbic activations were found in cocaine abusers compared with HCs during sexually arousing stimuli (Garavan et al. 2000) and this may indicate a relatively low sensitivity to natural rewards in SAs, also referred to as reward deficiency (Blum et al. 2000). Strengths of the Garavan et al. (2000) study were its homogeneous sample size (cocaine freebase [crack] smokers only) and its elaborate design, video blocks being separated by a short visuospatial task as a distracter to reduce carry-over effects. In another study in cocaine abusers, Wexler et al. (2001) found higher ACC activity both preceding and following the onset of craving while watching a cocaine video, but not when watching happy and sad video tapes, compared to HCs. In addition, cocaine abusers showed lower activation in various prefrontal and temporal areas compared with HCs during the cocaine-cue video. In contrast to Childress et al. (1999), the authors concluded that there was a fundamental neurobiological difference between craving and normal emotional states, most probably due to an imbalance between limbic and prefrontal cortical activity. During craving, cocaine-dependent subjects showed greater activity than HCs in regions that were found to be active in HCs when viewing sad video tapes compared to happy tapes, suggesting a physiological link between cocaine cue-responses and normal dysphoric states rather than normal euphoric states (Wexler et al. 2001).

In smokers, greater activation was found after exposure to smoking-related images compared with neutral images in several limbic brain regions (part of the mesocorticolimbic dopamine (DA) reward pathway), as well as in regions part of the visuospatial attention circuitry, compared to HCs (Due et al. 2002). The authors suggest that the reward and visuospatial attention circuitry act in concert to increase and direct attention to potentially important stimuli, such as smoking stimuli in deprived smokers (Due et al. 2002). This study thus replicated findings of increased limbic activation during processing of cocaine cues. However, in comparison to the previous studies performed during craving in abstinent cocaine-dependent individuals, the findings from this study may additionally reflect the effects of craving during acute (nicotine) withdrawal, which might be different from the effects of craving during long-term abstinence.

David et al. (2005) failed to observe significant differences in overall brain activation in a small study with smokers, suggesting that the absence of whole-brain group differences was due to wide inter-individual variability in magnitude and location of activation, indicating the need for larger sample sizes. In a secondary ROI-analysis, greater ventral striatum/nucleus accumbens (VS/NcA) activation was in smokers, but, however, no correlation was found between NcA activation and self-reported craving, which might be due to a ceiling effect due to nicotine withdrawal during the study (David et al. 2007). Also, Okuyemi et al. (2006) found significant group (smokers vs. HCs) by condition (smoking vs. neutral) interaction effects in medial PFC, right lateral OFC, and bilateral VLPFC activation. Moreover, additional limbic activation was found in the subgroup of African-American smokers compared with Caucasian smokers, indicating differential involvement of brain areas in smoking-related cue-reactivity in different ethnic groups (Okuyemi et al. 2006). When introducing monetary rewards in a drug cue-reactivity task, ACC activation in cocaine abusers was found significantly lower than in HCs (Goldstein et al. 2009b). Rostroventral ACC activity during reward trials was correlated with task-induced craving and caudal-dorsal ACC activity during no-reward trials was inversely correlated with current cocaine use. The authors concluded that emotional aspects of the task modulated ACC activation patterns in proportion to substance use severity (Goldstein et al. 2009b) although they found no effect of word (neutral vs. drug-related) on ACC activity. In a recent study, Goudriaan et al. (2010) found brain response differences in smokers only when the subgroup with the highest scores on the Fagerstrom Test for Nicotine Dependence (FTND; mean score = 5.4) was compared with HCs. This subgroup showed significantly more activation in ventromedial (VM) PFC, rostral ACC, insula, and middle/superior temporal gyrus while watching smoking related pictures than the group of HCs or smokers with low FTND scores, and nicotine craving correlated with activation in left PFC and left amygdala. Finally, Wilcox et al. showed higher dorsolateral prefrontal and occipital activation during cocaine-related videos in cocaine users versus HC; there were no differences between the groups during food-related control videos (Wilcox et al. 2011). In addition, a resting state connectivity analyses showed less connectivity between bilateral OFC and striatum combined with more connectivity between these regions and posterior cingulated cortex/precuneus in cocaine users compared to HC, suggesting impaired motivational decision making in cocaine users (Wilcox et al. 2011).

Altogether, 29 studies on cue-reactivity in SAs were identified, with only 10 of these meeting inclusion criteria for the current review: six in cocaine abusers and four in nicotine-dependent subjects (see Table 2). Unfortunately, there were no studies on amphetamine, methamphetamine, ecstasy, or caffeine abuse.

Summarizing, seven studies reported higher activity of the limbic system in SAs versus HCs, presumably indicating conditioned cue-reactivity (Childress et al. 1999; Garavan et al. 2000; Wexler et al. 2001; Due et al. 2002; David et al. 2005; Okuyemi et al. 2006; Wilcox et al. 2011), and seven studies reported higher activity of the (dorsal) ACC and/or PFC in SAs versus HCs, presumably representing activation of control circuitry to regulate the over-extensive drive toward drug-related stimuli (Maas et al. 1998; Childress et al. 1999; Garavan et al. 2000; Wexler et al. 2001; David et al. 2005; Goudriaan et al. 2010; Wilcox et al. 2011). The observed limbic over-activation in seven of the nine studies (including VS/NcA and ventral tegmental area [VTA] activation) is consistent with the I-RISA model of drug abuse. The I-RISA model also postulates lower activation of the PFC in SAs versus HCs. However, whereas most studies found higher frontal activation in SAs (Maas et al. 1998; Garavan et al. 2000; Due et al. 2002; David et al. 2005; Okuyemi et al. 2006; Goudriaan et al. 2010), other studies found lower or no frontal activation differences of SAs compared with HCs (Childress et al. 1999; Wexler et al. 2001). A possible explanation for these inconsistent findings might be that low PFC activation is due to overall reduced functioning in SAs compared with HCs, whereas high activation may reflect compensatory activity (resulting in similar behavioral responses between SAs and HCs), or increased cognitive control to block feelings of craving in SAs compared with HCs.

Despite the fact that some findings were replicated, the current review also shows a large variability between studies. In some cue-reactivity studies, SAs displayed lower ACC activation than HCs when faced with cue-related stimuli (Maas et al. 1998; Childress et al. 1999; Garavan et al. 2000; Wexler et al. 2001; Goudriaan et al. 2010), while other studies failed to replicate this finding (Due et al. 2002; David et al. 2005; Okuyemi et al. 2006; Wilcox et al. 2011), or even found lower activity of the ACC in SAs compared with HCs when presenting drug-related stimuli (Goldstein et al. 2009b). Differences in task and study design may have contributed to these different results (see Table 2). For example, all studies reporting increased ACC activity were performed in cocaine-dependent individuals while watching audiovisual/video materials, whereas studies that failed to observe altered ACC activity were mostly performed in smokers and used pictures during scanning sessions. Therefore, the lack of ACC activity in smokers might be related to the nature of the cues or to the abused substance. One study reported an association between regional brain activity and FTND scores (Goudriaan et al. 2010), showing that higher activation of VM PFC, rostral ACC, insula, and middle/superior temporal gyrus only occurred in heavy smokers with relatively high FTND scores compared with HCs. In addition, the only study reporting lower ACC activity used a complex design, with a drug Stroop task coupled with monetary rewards (Goldstein et al. 2009b). In this study, low ACC activation was observed primarily during presentation of neutral words during the no-reward condition, and is therefore difficult to compare with high ACC activity observed studies employing straightforward cue-exposure designs. Moreover, the study of Okuyemi et al. (2006) suggests that ethnic variation may lead to different results even when the same tasks and designs are used. Together, these sources of variation are likely to explain inconsistent findings in ACC activity in cue-reactivity paradigms.

Concerning brain regions of importance, both ACC and PFC are known to be involved when faced with complex and conflicting information and, subsequently, in social conflict resolution (Zaki et al. 2010). In addition, neurons of the dorsal ACC process information regarding both reward (magnitude and expectancy) and action (Shidara and Richmond 2002; Hayden and Platt 2010). Interestingly, in drug dependence, older studies found that lesions in ACC may reduce drug taking (Sharma 1974; Kanaka and Balasubramaniam 1978), which might explain the high ACC activation in SAs. The amygdala is known to process motivationally significant stimuli, but is also involved in active fear extinction and reinforcer devaluation (Morrison and Salzman 2010), while the anterior cingulate activates during conflict resolution (Zaki et al. 2010), for example, in abstinent drug-dependent individuals when faced with drug-related stimuli. The NcA is part of the cortico-striato-thalamo-cortical loop, and is important in drug-induced reinstatement of drug-seeking behavior. In addition, the NcA is prone to synaptic plasticity changes following drug use (Chen et al. 2010; Li et al. 2010; Russo et al. 2010). Furthermore, this brain area features prominently in drug addiction studies, and it has been hypothesized that the amount of striatal DA receptors may predict the predisposition or development of addiction (Nader et al. 2006; Piray et al. 2010). Many studies have replicated findings of increased limbic activation during processing of cocaine cues, which includes activation of the hippocampus, VTA, and thalamus, establishing the importance of the reward circuitry and the role of distinct brain memory systems in the encoding and retrieval of drug-related memories in drug-dependent individuals (Robbins et al. 2008; Sun et al. 2010). In correspondence with the I-RISA model, consistent findings of limbic dysregulation in SAs were found during cue-reactivity imaging, which probably reflects altered valuation of drug rewards.

### Section 3: Impulsivity in stimulant dependence

#### Task paradigms and behavioral findings of impulsivity

Impulsivity is a multi-domain concept involving several independent aspects, and thus has no unique neurological basis (Evenden 1999). Impulsivity has at least two major components: motor impulsivity (impulsive action or disinhibition), and cognitive impulsivity (impulsive choice). Both aspects are associated with the hallmarks of drug dependence according to *DSM-IV*: taking the substance longer and more often than originally intended; unsuccessful efforts to cut down or control drug intake; and spending more time and effort to obtain the drug (American Psychiatric Association 1994). High impulsivity levels are commonly associated with drug dependence and are postulated to underlie the etiology as well as the continuation of drug dependence (Adinoff et al. 2007; Verdejo-Garcia et al. 2008; Crews and Boettiger 2009; Wit 2009). In addition, motor and cognitive impulsivity are often correlated with relapse (Moeller et al. 2001; Adinoff et al. 2007).

##### Motor impulsivity

Adequate inhibitory control allows an individual to stop a premature, poorly conceived, and potentially risky response which would ultimately result in an undesired outcome (Evenden 1999). Adequate inhibitory control can thus be viewed as action error-monitoring of responses, and motor inhibition is necessary to ensure adaptive behavior with positive long-term outcomes. Stimulant dependence has been repeatedly associated with high motor impulsivity or a lack of inhibition (Evenden 1999; Fillmore and Rush 2002; Fillmore et al. 2002, 2003; Morgan et al. 2006; Quednow et al. 2007; Verdejo-Garcia et al. 2008) contributing to loss of control over drug use and excessive drug-taking behavior (Lyvers 1998).

The most common objective measures for motor inhibition are the Stop-Signal task (Logan et al. 1984), the Circle Tracing task (Bachorowski and Newman 1990), and the Go/No-go task. Whereas the difficulty of the Stop-Signal task involves stopping an already initiated response several milliseconds following a go-stimulus, the Go/No-go task measures impulse inhibition without a directly initiated response. These tasks require rapid, repeated target responses, while also demanding suppression of pre-potent or automated responses when faced with a stop or no-go stimulus. Performance can be characterized in terms of stop-signal reaction time (Stop Signal Task) and commission or omission errors (Go/No-go task). Commission errors are responses while a no-go target was presented and omission errors are nonresponses while a go target was presented. The Stroop task (see Section 2) can similarly be used to measure inhibition of an automated response, as this task requires suppression of an overlearned response (word reading) in favor of an atypical and hence effortful response (color naming). However, as discussed previously, this task additionally includes selective attention as cognitive process, making it more difficult to assess motor inhibition unrelated to cognitive interference components.

In a study using a Stop-Signal task, cocaine abusers showed reduced motor inhibition compared with HCs, and acute cocaine administration in cocaine abusers resulted in decreased inhibition compared with saline administration (Fillmore and Rush 2002; Fillmore et al. 2002). Using the Stroop task as a measure of motor impulsivity, no performance differences were found in male cocaine abusers compared to male HCs (Selby and Azrin 1998). Another study found a small (nonsignificant) decrement in performance during the Stroop task in abstinent cocaine abusers (Bolla et al. 1999). In adolescent smokers, performance on a Stroop task improved following smoking, whereas abstinence from smoking resulted in impaired inhibition (Zack et al. 2001).

##### Cognitive impulsivity

Cognitive impulsivity, or impaired delay discounting, constitutes an important aspect of decision making (Monterosso and Ainslie 1999; Cardinal et al. 2004; Deakin et al. 2004): inhibition of impulsive choosing behavior is important to make appropriate choices, for example, weighing the probability of short-term gains against the probability of long-term negative consequences. Specifically, impulsive choice making is characterized by a preference for obtaining small rewards now over large rewards in the future. In stimulant dependence, impulsive choice leads the individual to frequently terminate activities because they are not immediately gratifying (Evenden 1999). This may include relapse (to obtain an immediate rewarding effect) rather than staying abstinent, while being aware of longer term health benefits of abstinence.

Delay discounting tasks (DDTs) measure cognitive impulsivity by determining the individual's preference for an immediate small (monetary or drug) reward over a larger reward in the future. Using DDTs, some studies have shown that ecstasy use correlates with increased cognitive impulsivity (Morgan 1998; Oja et al. 2003; Quednow et al. 2007), which was still present during abstinence (Morgan et al. 2002b), whereas other studies failed to observe significant differences between ecstasy users and HCs (Hanson et al. 2008; Win et al. 2008). Methamphetamine-dependent abstinent individuals showed significantly higher delay discounting, indicating higher cognitive impulsivity, than HCs (Hoffman et al. 2006). Higher delay discounting for monetary rewards was also present in actively using and 30-day abstinent cocaine dependent individuals compared to HCs (Heil et al. 2006). In addition, higher delay discounting was found in cocaine-dependent patients compared with HCs for drug-related rewards compared to monetary rewards (Coffey et al. 2003). Smokers had higher discounting rates than nonsmoking controls when performing a DDT task with hypothetical money (Mitchell 1999; Reynolds et al. 2004), and this effect was even more robust when cigarettes or health outcomes were used as hypothetical rewards (Bickel et al. 1999; Baker et al. 2003). Finally, dosage and frequency of nicotine use in current smokers were correlated with levels of delay discounting for monetary rewards in smokers compared with nonsmokers (Ohmura et al. 2005).

#### Imaging studies on impulsivity: results and discussion

##### Imaging studies on motor impulsivity

Kaufmann et al. (2003) found smaller volumes of activation in the right DLPFC, the ACC, the inferior parietal lobule, and the putamen bilaterally in cocaine users compared with HCs. During both errors and successful no-go trials, activation was significantly lower in, for example, the ACC, proposing that an underactive action monitoring system in cocaine abusers may represent the neural correlate of compromised control over their (drug using) behavior (Kaufman et al. 2003). Given that active cocaine users were abstinent 18–72 h before testing, it is not possible to rule out acute withdrawal as a partial explanation of the findings. In addition, it should be noted that individual performance differences were not accounted for. Similarly, cocaine abusers exhibited lower activation in the ACC, presupplementary motor area (pre-SMA), and right PFC compared with HCs during correct inhibition trials in a similar study by Hester and Garavan (2004). A significant positive correlation between ACC activity and correct inhibition scores was found for the HCs, whereas ACC activity was unrelated to performance in cocaine abusers, hypothesizing that cocaine users have diminished ACC capacity to detect fluctuations in the need for inhibitory control, resulting in impaired implementation of inhibitory control and planning of motor actions through the (lateral) PFC and pre-SMA, respectively (Hester and Garavan 2004). In addition, in a more recent study, abstinent cocaine abusers showed significantly less activity in the rACC for successful over unsuccessful stop trials than HCs, and rACC activity was inversely correlated with scores on the impulsive subscale of the difficulties in emotion regulation scale (Li et al. 2008). Activation in the dmPFC did not differ between abstinent cocaine abusers and HCs, but was inversely correlated with mean stop signal reaction time (SSRT), concluding that low activity in the rACC was related to poor inhibitory control in abstinent cocaine abusers, whereas the dmPFC might be involved in response inhibition execution (Li et al. 2008). Using a Stroop task, Bolla et al. (2004) asked participants to correct each mistake before starting the next trial, to increase differences between conditions, and found that abstinent cocaine abusers showed less activation in the left caudal–dorsal ACC (midcingulate) and right lateral PFC, but stronger activation in the right ACC compared with HCs. Interestingly, activity in the right lateral PFC and the rostral–ventral ACC in cocaine abusers was negatively correlated with former average amount of cocaine used per week. The authors were thus able to only partially confirm their hypothesis that ACC and lateral PFC function is impaired in abstinent cocaine abusers compared with HCs, and suggested that the increased right ACC activation in cocaine abusers represents a compensatory mechanism (Bolla et al. 2004).

Although somewhat outside the scope of this review, two studies performing a robust motor task (finger tapping) rather than a specific motor inhibition task showed clear differences between psychostimulant abusers and HCs regarding motor performance, suggesting an association with increased motor impulsivity. While one study showed a significant association between motor performance deficits in chronic crack cocaine abusers and decreased activity in the dorsal striatum (Hanlon et al. 2009), another study found significantly more activation during tapping in the right SMA in MDMA users compared with HCs, and significant positive correlations were found between the number of MDMA episodes and activation in the right putamen and the right pallidum, and between lifetime episodes of MDMA use and the percentage of activated voxels in the right precentral cortex, thalamus bilaterally, and right postcentral cortex (Karageorgiou et al. 2009). The authors proposed that the increased SMA activation during the motor task might be due to a compensatory mechanism involving other brain regions afferent to SMA, an increased local synaptic activity or both, reflecting altered regional neurophysiology and being consistent with MDMA-induced alterations in the basal ganglia-thalamocortical circuit due to MDMA neurotoxicity, although additional research is warranted here (Karageorgiou et al. 2009).

To summarize, impaired response inhibition in cocaine users compared with HCs was reflected by lower activations in the (dorsal) ACC, lateral PFC, and pre-SMA. These findings are corroborated by a volumetric study showing decreased gray matter volume of the ACC in addition to superior temporal regions, and insula in cocaine users (Franklin et al. 2002), and a resting-state PET study showing decreased metabolic activity in the ACC and OFC (Volkow et al. 1993). This prefrontal dysregulation (decreased activity) is consistent with the I-RISA theory on the role of impaired response inhibition.

However, there is a clear need for functional imaging studies investigating inhibitory control in other stimulant addictions such as nicotine, (meth-)amphetamine, and caffeine use. A general methodological issue is that most studies published to date do not sufficiently control for the duration of abstinence (or time since last use). In addition, conflicting findings have been reported regarding rostral ACC, which was found to be less active in one study (Li et al. 2008) and more active in another study (Bolla et al. 2004). These discrepancies could be due to differences in imaging modalities or task paradigms (see Table 3).

##### Imaging studies on cognitive impulsivity

Methamphetamine-dependent users displayed higher delay discounting with difficult choices (i.e., choices close to the indifference point, where subjects are presumed to have equal preferences regarding immediate vs. delayed rewards) versus easy choices, resulting in lower activations of the left DLPFC and intraparietal sulcus (IPS) compared with HCs (Monterosso et al. 2007). However, no significant correlations between brain activation patterns and discounting rates were observed (Monterosso et al. 2007). In a study by Hoffman et al. (2008), abstinent methamphetamine users showed a significantly stronger preference for immediate rewards than HCs with lower activation in the precuneus and right caudate nucleus, ACC, and DLPFC. Here, low activation of the amygdala, DLPFC, posterior cingulate, and posterior parietal cortex was correlated with higher discounting rates. In addition, abstinent methamphetamine users exhibited more activation during easy choices and showed less activation differences between easy and difficult choices (Hoffman et al. 2008). Recently, Meade et al. (2011) found less activity in bilateral PFC and ACC during difficult versus easy choices in active cocaine users compared with HCs. In recovered cocaine users, activation patterns during easy choices were similar to those in HCs, but recovered users still revealed impairments during difficult choices (Meade et al. 2011).

Only three studies are available employing functional neuroimaging during DDTs in stimulant dependence, two of which were performed in methamphetamine abusers (see Table 3). Although one study was conducted in active users (Monterosso et al. 2007) and the other in abstinent abusers (Hoffman et al. 2008), similar brain areas were found to be less active in SAs compared with HCs for difficult versus easy choices. Similar results were obtained in active cocaine using HIV patients (Meade et al. 2011). These findings, therefore, indicate that, even after sustained abstinence, brain functions remain altered in methamphetamine and cocaine abusers, resulting in sustained periods with a high probability of relapse into drug use. In the methamphetamine studies, these group-by-task load effects were probably due to increased regional brain activity in methamphetamine users during “easy” choices, presumably reflecting lower efficiency of cognitive control circuitry. In contrast to Monterosso et al. (2007), Hoffman et al. (2008) observed significant correlations between discounting rates and activity in the DLPFC, amygdala, posterior cingulate cortex, and posterior parietal cortex. These latter findings are consistent with the hypothesis that both ventral/limbic and dorsal systems are involved in impulsive decisions: the ventral system (amygdala, ventral striatum, VLPFC, insula) for decisions involving salient and immediate rewards and the dorsal system (DLPFC, dorsal ACC, and posterior parietal cortex) when decision making requires elaborate comparison and choice making (McClure et al. 2004). Hoffman et al. (2008) suggested that their findings were consistent with a model wherein dorsal cognitive systems modulate the neural response of ventral regions. This switch from ventral to more dorsal striatal control is consistent with the hypothesis of a switch from salience-based behavior toward more habitual behavior and is linked with decreased sensitivity to outcome values (Habitual Behavioral Model). Indeed, methamphetamine-dependent patients, who strongly preferred smaller immediate over larger delayed rewards, appeared to activate the dorsal cognitive control system to overcome their preference for small immediate rewards. Moreover, activation of the amygdala during choice of delayed rewards was associated with a greater degree of discounting, suggesting that heavily discounting methamphetamine abusers may be more responsive to the negative salience of delayed rewards than controls. In contrast, in the Meade et al. (2011) study, differences in discounting rates, although in the expected direction, failed to reach statistical significance.

In conclusion, additional studies are warranted to elucidate the involvement of limbic regions compared with dorsal prefrontal areas in delayed discounting, and to better understand the dynamic interaction between the ventral (salience) and the dorsal (control) circuit. Whether similar changes can also be found in other stimulant abuse populations, such as cocaine, MDMA, nicotine, or caffeine abusers is still unknown.

### Section 4: Decision making and executive control in stimulant dependence

#### Task paradigms and behavioral findings of decision making and executive control

Decision making, memory, working memory, attention, cognitive flexibility, conflict monitoring, and planning are often conceptualized as separate elements of executive functioning, generally linked to intact (dorsal) PFC function (Smith and Jonides 1999; Funahashi 2001). In drug dependence, executive dysfunction may result in maladaptive decision making, preventing sound judgments regarding health benefits related to drug use, or cognitive inflexibility resulting in dependent individuals being unable to steer away from drug-related thoughts. Here we discuss task paradigms and behavioral findings regarding decision making, memory, and cognitive flexibility.

##### Decision making

Decision making can be assessed using the Iowa Gambling task (IGT) (Bechara et al. 1994) or a two-choice prediction task. The IGT stimulates the participant to gain money by turning cards of their choice from four virtual card decks: two containing large gains but even greater losses, and two decks with small rewards but even smaller losses. Thus, perseveration of risky choices will make the participant lose money. Using the IGT, methamphetamine and amphetamine abusers favored the risky high reward option (resulting in losses) compared with HCs (Rogers et al. 1999; Bechara et al. 2001). Moreover, decision-making speed and accuracy were impaired in amphetamine abusers and associated with duration of abuse, suggesting that repeated stimulant use may contribute to impaired decision making (Rogers et al. 1999). On the other hand, even small differences in decision-making strategies predicted future ecstasy use in ecstasy naive individuals (Schilt et al. 2009), implying a causal role for decision-making impairments in the development of stimulant abuse. Finally, in methadone-maintained abstinent heroin abusers, smokers showed impaired decision making during a gambling task as compared with nonsmokers (Rotheram-Fuller et al. 2004). The two-choice prediction task presents only two options: a risky option (high gains, but more losses) and a low-risk option (low gains, but few losses). The IGT and the two-choice prediction task are closely related to the PRLT discussed in Section 1, as they also involve positive and negative feedback. The IGT and the two-choice prediction task also address cognitive flexibility, which can also be measured using the Wisconsin Card Sorting Task (WCST) or the PRLT. However, the IGT and two-choice prediction task contain a more elaborate decision-making component (implement strategy and choice behavior that is advantageous in the long run vs. strategy and choice behavior that is disadvantageous in the long run) compared with the PRLT, and, therefore, we have chosen to discuss only the IGT and the two-choice prediction task in this section. Although the PRLT also comprises a decision making or choice component, the PRLT is not seen as a gambling task but a task measuring flexibility of learned behavior based on contingencies without the strategic element of long-term versus short-term advantages.

##### Memory

Immediate memory (and working memory: WM), is often assessed with the Wechsler Adult Intelligence Scale (WAIS) Digit Span or Memory Span task, requiring the person to remember a string of digits, letters or words. The N-back task is a continuous WM task which requires subjects to indicate whether the current letter matches the one from *n* (usually 1–3) steps earlier (Kirchner 1958). Delayed memory is addressed in the immediate memory task/delayed memory task (IMT/DMT), a task similar to the N-back task but with additional options (Dougherty et al. 2002), such as delaying the recognition phase up to several minutes. While these tasks mainly differ in the delay of the recognition phase, also the memory load differs in several tasks. For example, in the N-back task, working memory load can be increased by incorporating more steps back to be remembered in a short-time period, while the IMT/DMT can increase working memory load during a longer time period up to several minutes according to the task's design. Memory span tasks can also be made more challenging (increasing working memory load), that is, by instructing the individual to name the memory sets backwards. The WAIS digit span is similar to other memory span tasks, but is part of the more comprehensive full WAIS measuring both verbal intelligence quotient (IQ) and performance IQ. During a WM span task, male smokers performed worse than nonsmoking male HCs (Greenstein and Kassel 2009). Ecstasy users performed worse than HCs on a verbal DMT, and total ecstasy use was negatively associated with memory performance (Schilt et al. 2008). On a delayed memory recognition task, administration of a nicotine patch improved performance accuracy in nonsmokers (Froeliger et al. 2009). With regard to acute abstinence effects, in male smokers, memory performance declined across a 60-min test period, whereas aspects of calculation and association tasks improved over time (Sakurai and Kanazawa 2002).

##### Cognitive flexibility, attention, and planning

Attention is a complex process that can be divided in different aspects. For instance, sustained attention is the ability to maintain attention for a longer period on a certain task which can be measured using a sustained attention task, whereas divided attention is the ability to shift attention between different task demands. Cognitive flexibility or “set-shifting” is the ability to shift cognitive set depending on task demands (e.g., feedback) and is often assessed using the WCST (Grant and Berg 1948), which requires subjects to match cards following an unknown matching rule. Regardless of the unknown matching rule, the participant is told whether a match is correct or incorrect, and this task assesses the participant's flexibility to shift toward new responses. Whereas switch tasks are usually simplified tasks demanding cognitive flexibility including a switch that is explicitly mentioned during task instructions (explicit switching), the WCST comprises an implicit switch which the individual has to learn based on received feedback during the task. Ecstasy users performed worse on a variety of behavioral tasks including attention and perceptual organization compared with HCs (for a systematic review, see Rogers et al. 2009). Also, both cocaine and methamphetamine abusers performed significantly worse than HCs on measures of cognitive flexibility (WCST; Plas et al. 2008). In a study in recreational polydrug cocaine users, cognitive flexibility, but not WM, was found to be impaired compared with HCs (Colzato et al. 2009). Finally, ecstasy users performed worse than HCs on cognitive flexibility as assessed by the WCST and on a verbal DMT (Smith et al. 2006). In poly-substance (cocaine, methamphetamine, and alcohol) abusers, impaired WM and cognitive flexibility was found compared with HCs (Verdejo-Garcia et al. 2006).

Planning ability is often measured using the Tower of London (ToL) (Krikorian et al. 1994) or the very similar Stockings of Cambridge test, both tasks requiring the participant to solve a problem in as few steps as possible. Both tests measure identical processes, with the only difference between them being that the Stockings of Cambridge test is part of a larger copyrighted test battery, the CANTAB. Sleep-deprived participants receiving a dose of dexamphetamine performed the ToL for planning ability in significantly fewer moves, whereas subjects receiving caffeine performed significantly worse on the ToL compared with participants on placebo (Killgore et al. 2009).

#### Imaging studies on decision making and executive control: results and conclusions

##### Decision making

Using the IGT, abstinent cocaine abusers showed greater activation in the right OFC, left putamen, and left postcentral gyrus than HCs and lower activation compared with controls in right DLPFC, superior parietal lobule, left medial PFC, and right cerebellum compared with HCs (Bolla et al. 2003). Also, successful decision strategies (resulting in more wins and fewer losses) were correlated with higher OFC activity in both groups, and the amount of cocaine used before abstinence correlated negatively with left OFC activity in the cocaine users. It should be noted, however, that although [^15^O] PET is not prone to susceptibility artifacts in the OFC which can be problematic when using fMRI, the temporal resolution of [^15^O] PET is limited, permitting block designs only. Therefore, group differences regarding specific events (e.g., gain and loss trials) could not be assessed in this study, which awaits replication using an fMRI event-related design. In a [^15^O] PET study by Ersche et al. (2005) amphetamine abusers, one-year abstinent amphetamine/opiate abusers, and HCs showed no significant differences in task performance, but HCs showed greater activation in the right DLPFC, whereas current and abstinent amphetamine users showed greater activation in the left OFC as compared with HCs. Apart from the methodological issues regarding [^15^O] PET, this latter study is particularly interesting because their decision-making task was specifically designed to exclude the possible confounding effects of differences in working memory load and visuomotor demands and because the task excluded the learning component (Ersche et al. 2005).

In a study by Paulus et al. (2003), activation of the OFC, DLPFC, ACC, and parietal cortex was associated with success rates in HCs, while frontal activation in methamphetamine users was found irrespective of success, and activation of the OFC, DLPFC, and parietal cortex was highest when outcome was most unpredictable. According to the authors, these findings did not support the hypothesis that methamphetamine abusers are less sensitive to success or failure than HCs, but rather suggest an altered top-down modulation of response selection during decision making (Paulus et al. 2003).

In summary, two studies on decision making showed decreased DLPFC activation in SAs (Bolla et al. 2003; Ersche et al. 2005) coupled with increased activations in the OFC, parietal cortex, putamen, and the postcentral gyrus, whereas another study showed increased activation in the DLPFC in SAs compared with HCs (Paulus et al. 2003). A possible explanation for this discrepancy may be the use of different tasks: IGT (Bolla et al. 2003), Cambridge Risk Task (Ersche et al. 2005) or two-choice prediction task (Paulus et al. 2003; see Table 4). It should be noted that decision-making paradigms as currently employed are complex tasks, covering many aspects of decision making, including attention, WM load, and learning processes. Future studies need to differentiate between these various aspects, for example, by including specific control conditions, to delineate the brain circuitry involved in different aspects of decision making in SAs and HCs.

##### Immediate and delayed memory

Ecstasy users demonstrated larger activation in the right parietal cortex during the 1 and 2 back condition of an N-back task, and lower activation in frontal and temporal areas (the left superior temporal lobe, the left superior frontal gyrus and the ACC) during the 2-back condition (Daumann et al. 2003a). As the ecstasy users showed slightly longer RTs when performing the 2-back condition, it is suggested that differences in motivational aspects or cognitive strategies might underlie the activation differences (Daumann et al. 2003a). When repeating the study with three groups, ecstasy-only users, polyvalent ecstasy users, and HCs, again no performance differences were found between users and HCs (Daumann et al. 2003b). It should be noted, however, that seven of the eight ecstasy users were also included in this previous study. Increased task load was correlated with increased activation in the premotor cortex and was again associated with smaller activations in inferior temporal regions in pure ecstasy users compared with HCs (Daumann et al. 2003b). In addition, when comparing ecstasy-only users with polyvalent ecstasy users, lower activation was found in the angular gyrus and the striate cortex, suggesting that ecstasy use, and not concomitant use of other drugs, was responsible for the specific abnormalities found in ecstasy users (Daumann et al. 2003b). As no performance differences were present, interpretation of these imaging results is somewhat problematic, because the possibility of ceiling effects cannot be ruled out. In a small N-back study by Jacobsen et al. (2004), left hippocampus deactivation was observed in HCs, but not in ecstasy users, an effect that was especially noticeable during high WM load and was negatively correlated with time since last ecstasy use. The authors hypothesized that left hippocampal activity might be associated with working memory deficits found in ecstasy users (Fox et al. 2001; Reneman et al. 2001), and that this may recover with sustained abstinence, as suggested by the inverse relationship between hippocampal activation and duration of abstinence. However, in view of the small sample sizes and the established role of the hippocampus in episodic rather than working memory, this study is clearly in need of replication. Moreover, altered activation of the left hippocampus is probably due to the neurotoxic effect of ecstasy on serotonergic neurons that modulate inhibitory circuits in the hippocampus, which is in line with studies showing reduced glucose metabolism in the left hippocampus of adult ecstasy users (Buchert et al. 2001; Jacobsen et al. 2004). Given that hippocampal involvement is a common feature of resting-state network activity, one may question the specificity of these findings (Damoiseaux et al. 2006). In a more recent N-back fMRI study, Bustamante et al. (2011) found similar task performance between cocaine-dependent males and HCs, but the cocaine group showed less activity in the left inferior parietal cortex compared with HCs. The authors suggested that decreased parietal activity might reflect cocaine-induced attentional deficits, although this explanation is not easy to reconcile with intact performance as observed in their study.

In summary, during WM tasks performed in ecstasy and cocaine users compared with HCs, activation differences were found in frontal, parietal, and temporal areas, ACC, and left hippocampus, in the absence of performance differences. This discrepancy may be due to ceiling effects in task performance or to the fact that regional brain activity as measured using fMRI is more sensitive. To date, no neuroimaging studies comparing SAs and HCs on delayed memory have been published, nor have any addiction models included hypotheses toward memory deficits in addicted individuals, making it difficult to interpret these results in light of the current models of drug addiction.

##### Cognitive flexibility, attention, and planning

In a switching task, cocaine users showed decreased activation in the left cingulate gyrus, medial and right middle frontal gyrus, left thalamus, lentiform nucleus (globus pallidus/putamen), and right precuneus compared with HCs (Kubler et al. 2005). However, activation in the DLPFC and anterior frontal cortex was similar in both groups. The authors concluded that the diminished responsiveness in anterior cingulate and prefrontal areas is in concordance with the hypothesis of under-responsive action monitoring in cocaine abusers, and that cocaine users are selectively impaired for attention switching within WM, so that, for example, steering away from drug-related thoughts is problematic (Kubler et al. 2005). This study is of interest because it is the only study assessing both verbal and visuospatial WM switching in cocaine abusers compared with HCs, showing specific impairment in visuospatial WM in cocaine abusers. Using a PRLT, HCs showed higher activation of the ventrolateral PFC and premotor area than smokers during reversals following monetary loss (de Ruiter et al. 2009). However, smokers (compared with HCs) showed higher activation in the right insula and frontal operculum during reversal after monetary loss. In this, cognitive flexibility in smokers was affected but planning was intact. Smokers were asked to abstain from smoking 10 h before scanning. This may have interfered with performance and/or BOLD-activation due to withdrawal effects. However, the authors argue that this is unlikely given the intact planning in smokers. Finally, a study by Goldstein et al. (2007b), investigating practice effects (habituation) on a sustained attention task, showed a decrease in activation of the ACC, frontal areas, and cerebellum as compared with HCs, which was associated with measures of craving, frequency of use, and length of abstinence in cocaine users versus HCs. These findings are somewhat surprising as decreased prefrontal activation during prolonged or repeated task performance is usually considered to reflect increased neural efficiency, due to, for example, absence of novelty effects. In addition, cuneus and precuneus were more active in HCs as compared with cocaine abusers, and signal decreases in the thalamus correlated with RT decreases related to practice sessions, especially in cocaine abusers as compared with HCs (Goldstein et al. 2007b), hypothesized to reflect a changed ability to adapt to previously experienced situations as compared with HCs.

de Ruiter et al. (2009) assessed planning ability in smokers and HCs, but did not find differences in activation patterns, with the exception of a right posterior parietal area which was more active in HCs than in smokers.

Overall, very few functional imaging studies were available on cognitive flexibility (see Table 4). While SAs (cocaine-dependent subjects) showed decreased activation during a cognitive flexibility task in the anterior cingulate gyrus, medial PFC, and subcortical regions (thalamus and lentiform nucleus), no differences were found in lateral prefrontal cortices (DL and anterior frontal) compared with HCs. During an attention task, however, decreased DL (and VM) PFC as well as ACC, and medial frontal gyrus activation was found in SAs (cocaine) compared with HCs, but activation patterns between smokers and HCs did not differ during planning.

## General Discussion

A number of converging findings emerged in key brain regions during specific tasks, including increased activation in the limbic system following cue-reactivity paradigms, and increased DLPFC and PFC activity in cognitive and motor impulsivity studies, respectively. However, there were also several inconsistencies, which can probably be explained by methodological differences with regard to tasks and protocols used, study population, imaging modalities, and data analysis. Whereas we discussed these possible explanations in each section separately, in this section we will discuss some general issues in neuroimaging research and provide an outline for future research. Unfortunately, as mentioned before, only few studies are available on executive functioning, precluding assessment of common findings and inconsistencies in these areas. Also, two previous reviews concluded that there was reduced anterior and posterior cingulate activation, and reduced inferior frontal, DLPFC, and parietal activation during process-related functioning, but these studies were limited to cocaine and (meth-)amphetamine users (Hong et al. 2009; Gu et al. 2010). Both reviews are very similar in their conclusions regarding differences between users and controls: both proposed that altered brain activation patterns are related to the demand-specific processing of information, rather than generic differences between stimulants users and controls. In addition, both reviews also conclude that these differences are consistent with a shift to more stereotyped, habitual behavior.

The findings of this review appear to fit rather well a number of aspects of different but partly overlapping theories of drug addiction. Reward and punishment-, motor impulsivity-, and cue-reactivity imaging studies support a role for the I-RISA model: impaired prefrontal functioning that may play a key role in inadequate evaluation of natural reinforcers and in impaired response inhibition, while limbic dysregulation (e.g., amygdala overactivation) would reflect increased valuation of drug stimuli. Together, impaired prefrontal activity and overactivation of limbic structures would thus result in maladaptive (impulsive and compulsive) behaviors and risky sensations (craving) leading to persistent drug use and relapse into recurrent episodes of maladaptive drug use with long-term negative consequences. Aspects of the Incentive-Sensitization theory, with its emphasis on neurobiological changes paving the way to more persistent drug use, can only be tested in longitudinal/prospective studies of drug users and HCs, that are currently not available, but it stresses the important role of neurobiological changes in areas such as the dorsal PFC, dorsal ACC, and various limbic structures, that is, increased or decreased responsiveness of brain circuits, related to the repeatedly observed changes in the function of these brain areas (regardless of cause or effect). Finally, the Habitual Behavioral Model stresses impulsivity and decision related changes that were observed in the dorsal system (DLPFC, dorsal ACC, and posterior parietal cortex) and the more ventral regions (amygdala, ventral striatum, VLPFC, insula). However, with the exception of a single study (Wilcox et al. 2011), no analyses of functional connectivity were found in the reviewed articles, a limitation when considering pathways supposedly involved in addiction as presented in several articles. For example, recent studies using a resting state approach have shown lowered connectivity between VTA and thalamus/NcA related to years of drug use in chronic cocaine dependent individuals (Gu et al. 2010) and for disrupted dorsal anterior cingulate and ventral striatum/extended amygdala pathways in nicotine dependent individuals (Hong et al. 2009, 2010). Similarly, a recent resting state fMRI study showed a reduction in brain connectivity in prefrontal hemispheres in abstinent cocaine abusers relative to HCs (Kelly et al. 2011). More specifically, this study showed a relation between chronic cocaine dependence and reduced connectivity in a dorsal frontoparietal network involving the lateral frontal, medial premotor, and posterior parietal areas, indicating an impaired attentional network in cocaine users compared with HCs (Kelly et al. 2011). However, these studies are beyond the scope of the current review because they lack a HC group and/or do not use a neurocognitive task.

As was discussed previously, inconsistencies in findings may at least partly be explained by methodological heterogeneity, stressing the need for similar neurocognitive tasks and experimental procedures. Neurocognitive tasks are continuously altered and improved to meet new research questions. Such continuous modifications, while helpful for further research, also limit comparability across studies, which is problematic when only a limited number of studies in SAs with HCs are available. Note also that test–retest reliability for fMRI designs such as reward paradigms, while acceptable at a group level, are moderate at best for single subjects (Fliessbach et al. 2010). In addition, tasks often do not identify separate components of neurocognitive functioning. For example, most decision-making tasks do not only measure the process of decision making, but also processes related to attention, WM, reward expectation, and reward and punishment processing. Identifying these separate components of, for example, decision making may also be achieved by including carefully selected control tasks.

In addition to these issues related to task paradigms, differences in fMRI data acquisition and analysis are likely to be another major source of discrepancies across studies. As discussed previously, studies may differ with regard to scanner type, field strength, acquisition parameters, and data modeling (e.g., block vs. event-related). More generally, the BOLD fMRI technique has several limitations, such as susceptibility to signal distortion and dropout in the vicinity of bone-air transitions, such as the nasal sinuses, resulting in poor sensitivity to detect activity in, for example, medial OFC. Also, while BOLD fMRI is predicated on the assumption of increased regional perfusion being associated with greater neural activity, this neurovascular coupling may be compromised in elderly people but also following drug intake (Schwarz et al. 2007). Finally, the use of various data analysis techniques and (the massive number of) statistical tests can also be an important source of variation. Ideally, greater weight should be given to studies in which type I error is adequately controlled for, either by using whole-brain corrections for multiple testing or the use of independently derived a priori (as opposed to post hoc) ROIs. Some of the described studies have used various types of corrections (for whole-brain analyses [Daumann et al. 2003b; Okuyemi et al. 2006; Karageorgiou et al. 2009], multiple testing [Paulus et al. 2003; Bolla et al. 2004; Hester and Garavan 2004; Ersche et al. 2005; Kubler et al. 2005; Hoffman et al. 2006; Goldstein et al. 2007b; Li et al. 2008; Hanlon et al. 2009; de Ruiter et al. 2009], or pre-defined ROI analyses [Maas et al. 1998; Due et al. 2002; Bolla et al. 2004; Jacobsen et al. 2004; Ersche et al. 2005; Okuyemi et al. 2006; Li et al. 2008; Karageorgiou et al. 2009]) to reduce possible type I errors. However, only a limited number of these have controlled adequately for type I errors (Ersche et al. 2005; Okuyemi et al. 2006; Karageorgiou et al. 2009), and results from these studies should receive greater weight. Other studies used no (Childress et al. 1999) or inadequate (Bolla et al. 2003; Daumann et al. 2003a; Goldstein et al. 2007b) corrections, or did not provide information on this issue (Garavan et al. 2000; Wexler et al. 2001; Kaufman et al. 2003; David et al. 2005; Monterosso et al. 2007; Goldstein et al. 2009b), making it difficult to exclude possible false positive findings.

A final issue concerns interpretation of results, in particular with regard to behavioral and neurophysiological (BOLD) data. In studies in which similar performance on neurocognitive tasks was observed between HCs and SAs, or task specifics were manipulated to obtain similar performances, differences in regional activations are usually explained by some kind of compensation hypothesis, stating that higher activations coupled with similar performance may result from decreased neural efficiency coupled with compensatory mechanisms, so-called “compensatory scaffolding” (Park and Reuter-Lorenz 2009). However, subjects whose scaffolding capacity is limited, such as older adults, reach their resource limitations at lower levels of task demand (compensation-related utilization of neural circuits [CRUNCH] hypothesis). Also, the use of alternative cognitive strategies may necessitate recruitment of additional neuronal systems (Noppeney et al. 2004). In contrast, impaired behavioral performance in the presence of lower activation is generally interpreted as a malfunctioning circuit without sufficient compensation. Within this framework, normal performance coupled with decreased BOLD-responsiveness in patients is difficult to account for, although some authors have proposed increased baseline activity as an explanation (Wexler et al. 2001). However, to test this hypothesis, study designs should include both a high-level and a low-level baseline (e.g., Canli et al. 2005), but to our knowledge such studies in addiction disorders have not been published.

It is important to realize that this review compares substrates of neuropsychological functioning across a variety of different stimulant drugs. In general, future studies should be performed to investigate whether similar changes are to be found for all psychostimulant drugs (nicotine, [meth-]amphetamine, ecstasy, caffeine) and possibly also for other psychotropic drugs (opiates, alcohol, cannabis) and for behavioral addictions (e.g., de Ruiter et al. 2009). Such studies should employ similar or even identical tasks, and use similar statistical approaches and significance thresholds. In addition, future studies should attempt to control for variability in gender ratio, time of abstinence, duration and amount of drug use, time of onset of drug use/abuse, and polydrug use. In addition to these methodological issues, such studies should attempt to examine separate components of neuropsychological functions, rather than using tasks that address broad cognitive functions. Alternatively, future studies may vary task load, for example, by employing a parametric design, which may also be useful to avoid bottom and/or ceiling effects with regard to task performance. Finally, future studies should not compare SAs only to HCs, but also to other drug users (e.g., alcohol or opiods dependent subjects) or patients with other psychiatric disorders (e.g., ADHD or obsessive–compulsive disorders) to investigate the specificity of any findings, and to explore common abnormalities in different categories of disorders. A final promising development for future research is the combination of functional neuroimaging with pharmacological challenges to test the potential usefulness of certain compounds for the treatment of stimulant dependence, and to gain better insight in the neuropharmacological correlates of stimulant dependence.

## Conclusions

This review has both strengths and limitations. The main strengths include the careful selection of studies including only papers with SAs and HCs, the thematic ordering of the studies using integrated addiction models as the organizing principle, and the detailed description of the study populations and the tasks that were used in the selected studies. The review also has limitations. First, although we aimed to exclude studies in polysubstance users, most SAs were also smokers so that effects of nicotine could not be excluded. Second, many studies failed to adequately report the duration of substance use, so that correlations between abuse duration and morphological and functional brain abnormalities could not be assessed. Third, gender distribution was often unequal in the study groups, which is likely to be relevant because significant sex differences have been found in brain responses in HCs as well as in patients with stimulant dependence (Goldstein et al. 2005; Li et al. 2005; Adinoff et al. 2006). However, we chose not to exclude studies performed in mixed male and female samples, because only five studies included males only (Maas et al. 1998; Childress et al. 1999; Li et al. 2008; de Ruiter et al. 2009; Goudriaan et al. 2010). Finally, although some neuroimaging studies are available for pharmacological effects of caffeine (Liau et al. 2008; Perthen et al. 2008; Addicott et al. 2009) and for neurocognitive functioning following (nonexcessive) caffeine consumption (Portas et al. 1998; Bendlin et al. 2007; Koppelstaetter et al. 2008, 2010), to date studies on heavy caffeine intake compared with no caffeine using subjects have not been published.

The findings in this review are potentially important in the development of new interventions for the treatment of patients with a stimulant use disorder as both existing and novel neuromodulation techniques are currently implemented and tested in addiction treatment settings. Existing techniques include EEG neurofeedback (e.g., Sokhadze et al. 2008) and rTMS (Feil and Zangen 2010), whereas novel techniques include real-time fMRI neurofeedback (e.g., deCharms et al. 2005) and deep brain stimulation (e.g., Kuhn et al. 2007; Zhou et al. 2011). To select the most promising target regions for these interventions, robust data on the functional differences between SAs and HCs are of utmost importance, including knowledge about the direction of the differences between patients and HCs. The current review adds to our knowledge about the most robust observational findings and the most promising targets for these interventions.
